# Insights into the mechanism of membrane pyrophosphatases by combining experiment and computer simulation

**DOI:** 10.1063/1.4978038

**Published:** 2017-03-03

**Authors:** Nita R. Shah, Craig Wilkinson, Steven P. D. Harborne, Ainoleena Turku, Kun-Mou Li, Yuh-Ju Sun, Sarah Harris, Adrian Goldman

**Affiliations:** 1 School of Biomedical Sciences and Astbury Centre for Structural Molecular Biology, University of Leeds, Leeds, United Kingdom; 2 Division of Pharmaceutical Chemistry and Technology, Faculty of Pharmacy, University of Helsinki, Helsinki, Finland; 3 Department of Life Sciences and Institute of Bioinformatics and Structural Biology, College of Life Sciences, National Tsing Hua University, Hsinchu 30013, Taiwan; 4 School of Physics and Astronomy and Astbury Centre for Structural Molecular Biology, University of Leeds, Leeds, United Kingdom; 5 Division of Biochemistry, University of Helsinki, FIN-00014 Helsinki, Finland

## Abstract

Membrane-integral pyrophosphatases (mPPases) couple the hydrolysis of pyrophosphate (PP_i_) to the pumping of Na^+^, H^+^, or both these ions across a membrane. Recently solved structures of the Na^+^-pumping *Thermotoga maritima* mPPase (TmPPase) and H^+^-pumping *Vigna radiata* mPPase revealed the basis of ion selectivity between these enzymes and provided evidence for the mechanisms of substrate hydrolysis and ion-pumping. Our atomistic molecular dynamics (MD) simulations of TmPPase demonstrate that loop 5–6 is mobile in the absence of the substrate or substrate-analogue bound to the active site, explaining the lack of electron density for this loop in resting state structures. Furthermore, creating an *apo* model of TmPPase by removing ligands from the TmPPase:IDP:Na structure in MD simulations resulted in increased dynamics in loop 5–6, which results in this loop moving to uncover the active site, suggesting that interactions between loop 5–6 and the imidodiphosphate and its associated Mg^2+^ are important for holding a loop-closed conformation. We also provide further evidence for the transport-before-hydrolysis mechanism by showing that the non-hydrolyzable substrate analogue, methylene diphosphonate, induces low levels of proton pumping by VrPPase.

## I. INTRODUCTION

Three types of convergently evolved enzymes hydrolyze inorganic pyrophosphate (PP_i_): type I and type II soluble pyrophosphatases (sPPases) and membrane-integral pyrophosphatases (mPPases). PP_i_ is generated by at least 190 cellular reactions including DNA biosynthesis and aminoacyl-tRNA generation; thus, tight control of PP_i_ levels in the cell is crucial to prevent product inhibition of these reactions.^1–5^ sPPases are present in all cells and are primarily responsible for managing cellular PP_i_ levels, with a *k*_cat_ of ∼200–2000 s^−1^.^6^ In contrast, mPPases are slower (*k*_cat_ of 3–20 s^−1^), only found in select species,^6^ and utilize the hydrolysis of PP_i_ to pump ions across a membrane, generating an electrochemical gradient.

Excluding multicellular animals, such as mammals, mPPases are found in organisms across all three domains of life. Plants and algae express mPPases in vacuolar membranes^7–9^ that, along with the vacuolar (V-type) H^+^-ATPase, acidify the organelle and are involved in development and stress resistance.^10–13^ mPPases are also found in the membranes of the acidocalcisome, a relatively small organelle found in protozoan parasites that plays a crucial role during the transition between environments with varying osmotic pressures.^14–16^ Furthermore, mPPases are present in many bacterial species, several of which are opportunistic human pathogens, such as members of genus *Bacteroides*.^17–19^ Overexpression of mPPases in bacteria confers resistance to heat, hydrogen peroxide, and salt stress.^20^

mPPases are selective in the ions they pump across the membrane: Na^+^, H^+^, or both (dual-pumping mPPases). Na^+^-pumping mPPases, thought to be the ancestral form of the enzyme, and dual-pumping mPPases occur in bacteria, with the former also found in archaea; both of these require potassium for optimal activity.^17,21^ Plants and protozoans, conversely, contain only H^+^-pumping mPPases.^8^ The H^+^-pumping mPPases are further subdivided into those that require potassium ions for optimal activity and those that do not (K^+^-dependent and K^+^-independent types, respectively). The difference is due to a key lysine residue (K^12.46^, using the Ballesteros and Weinstein numbering system, as previously described^22^) that can functionally replace K^+^ in the active site.^23^

Recently, structures of two mPPases, the Na^+^-pumping *Thermotoga maritima* mPPase (TmPPase) and the H^+^-pumping, K^+^-dependent *Vigna radiata* mPPase (VrPPase), have been solved in different conformations by X-ray crystallography.^24–26^ These static snapshots of different states through the catalytic cycle, such as the resting state and the substrate analogue-bound state, provide insight into the possible mechanism of hydrolysis and ion-pumping. However, proteins can sometimes be forced into non-native conformations due to crystallographic contacts, resulting in artefacts.^27^ We have generated atomistic molecular dynamics (MD) simulations of the membrane-integral TmPPase within a lipid bilayer that highlight the importance of interactions displayed in the crystal structures, thus strengthening evidence for our proposed model of the catalytic mechanism. In addition, we have recently provided experimental data that support the hypothesis that ion pumping precedes PP_i_ hydrolysis in mPPases,^26^ and here, we provide further experimental support for this mechanism of action.

### A. Overview of mPPase structures

TmPPase has been solved in the resting state (TmPPase:Ca:Mg; PDB: 4AV3),^24^ substrate analogue imidodiphosphate (IDP)-bound state (TmPPase:IDP:Na; PDB: 5LZQ),^26^ phosphate analogue tungstate (WO_4_)-bound state (TmPPase:WO_4_; PDB: 5LZR),^26^ and a product-bound state (TmPPase:P_i2_; PDB: 4AV6).^24^ VrPPase has been solved in a substrate analogue imidodiphosphate (IDP)-bound state (VrPPase:IDP; PDB: 4A01) and a single phosphate-bound state (VrPPase:Pi; PDB: 5GPJ).^25^

**FIG. 1.**
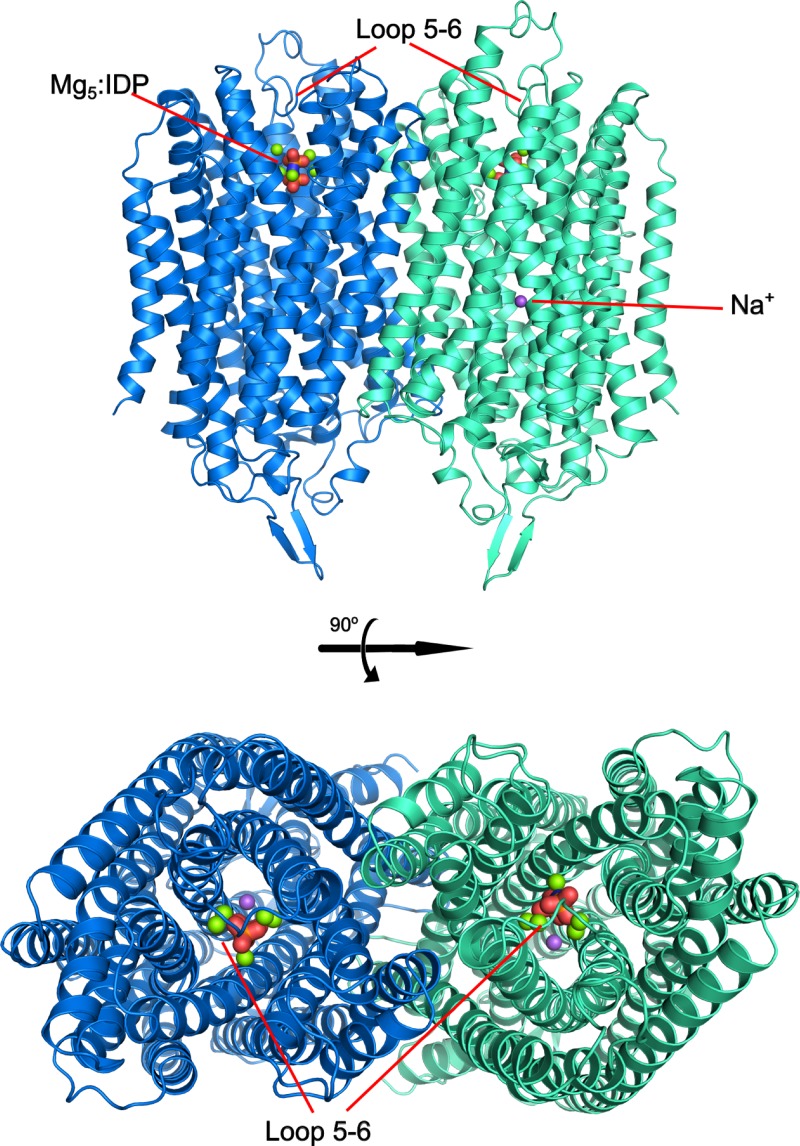
Overall protein structure of TmPPase in the substrate-analogue-bound state (PDB ID: 5LZQ). The protein is shown as a dimer with each protomer depicted in a different color. IDP is shown in red with Mg^2+^ and Na^+^ shown as green and purple spheres, respectively.

These structures reveal that TmPPase and VrPPase are, overall, very similar (Fig. 1);^24,25^ for instance, the Cα root-mean-square deviation (RMSD) of the IDP-bound states is only 0.862 Å for 725 aligned residues.

## II. RESULTS

### A. MD simulations support the mechanism of substrate binding and hydrolysis

**FIG. 2.**
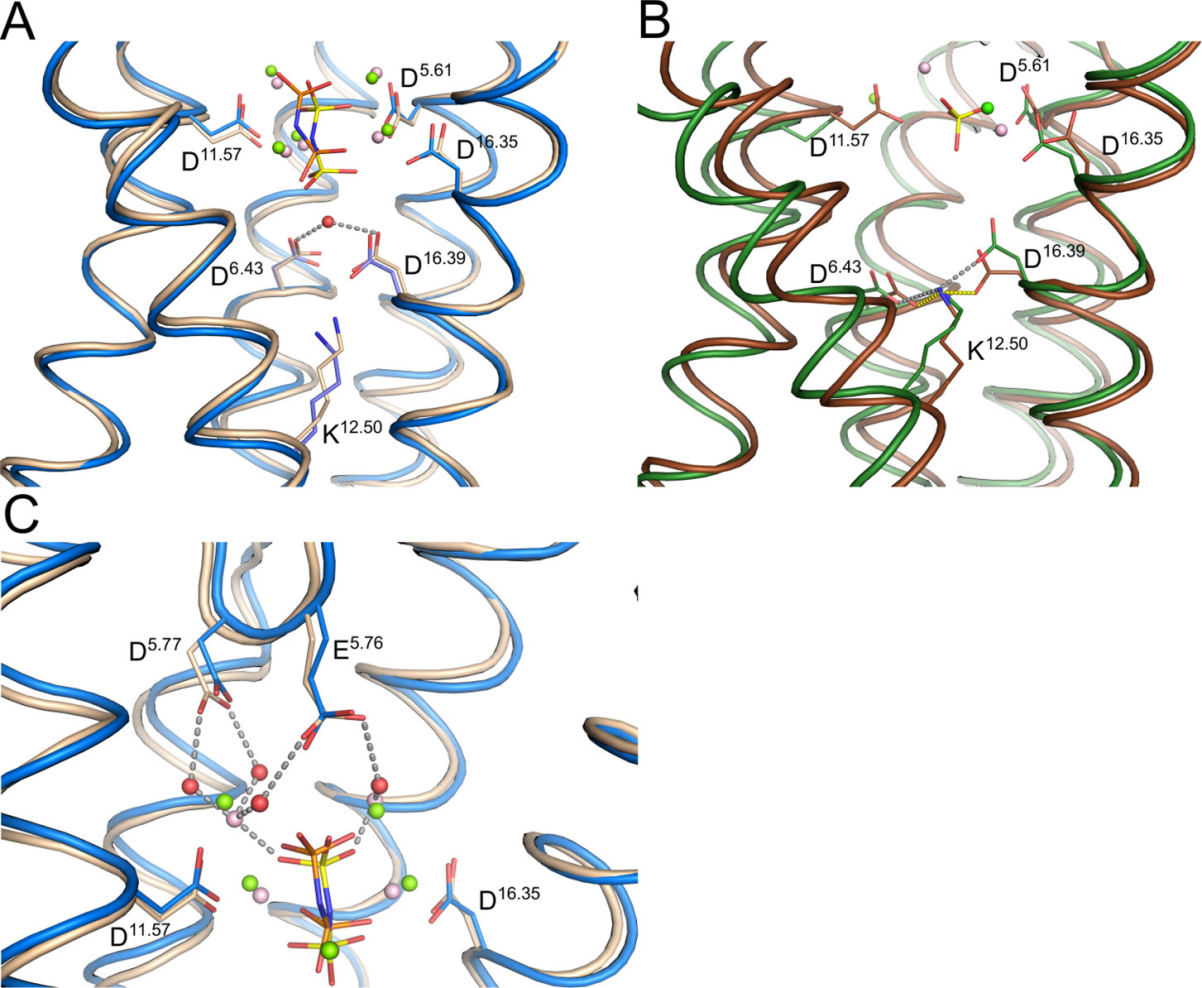
Structural overview of different regions of the mPPase hydrolytic region in different catalytic states. Comparison of the base of the hydrolytic center in (a) TmPPase:IDP:Na (blue) and VrPPase:IDP (light brown); and in (b) TmPPase:Ca:Mg (green) and VrPPase:P_i_ (brown). (c) Comparison of the cytosolic side of the hydrolytic center in TmPPase:IDP:Na and VrPPase:IDP. In all structures, water molecules are depicted as red spheres, including the nucleophilic water in (a). Mg^2+^ and Ca^2+^ are depicted as green spheres in TmPPase and salmon spheres in VrPPase. IDP and phosphate are depicted with orange phosphorous atoms for TmPPase structures and yellow for VrPPase structures.

The recently solved structures of mPPase have allowed us to build a comprehensive model for the mechanism of PP_i_ hydrolysis, which we believe occurs *via* activation of a nucleophilic water molecule. In both TmPPase:IDP:Na and VrPPase:IDP, the nucleophilic water is coordinated by D^6.43^, a conserved Asp on TMH 6, and D^16.39^, a conserved Asp on TMH 16 (Ballesteros and Weinstein numbering system^22^) (Fig. 2(a)). However, in the TmPPase resting structure (TmPPase:Ca:Mg) and the VrPPase single phosphate-bound structure (VrPPase:P_i_), D^6.43^ in particular, is coordinated by K^12.50^, a key Lys on TMH 12 (Fig. 2(b)). This interaction prevents it from coordinating the nucleophilic water by holding TMH 6 in a strained conformation, thus preventing enzyme activation. The structures also suggest that binding of IDP by both TmPPase and VrPPase leads to a closing of loop 5–6 (the loop between TMH 5 and 6) and loop 13–14 over the active site. A lack of electron density for loop 5–6 in the TmPPase resting state structure suggests that this loop is disordered in the resting state, but the electron density shows that loop 5–6 is closed over the active site in the substrate analogue-bound structures.^24–26^ Residues E^5.76^ and D^5.77^ on loop 5–6 in both VrPPase:IDP and TmPPase:IDP:Na interact with the substrate-analogue Mg_5_:IDP complex *via* water molecules in the hydrolytic center, which may contribute to the stability of the loop-closed conformation (Fig. 2(c)). We hypothesize that closing of these loops over the active site is a critical step in the catalytic cycle, coupling correct substrate binding to conformational changes that lead to hydrolysis. The downward movement of TMH 12 that is associated with loop-closure breaks the coordination of K^12.50^ with D^6.43^ and D^16.39^ and releases the torsional deformation of TMH 6, akin to a “molecular mousetrap.” This leads to a reorientation of TMH 6 and allows D^6.43^ and D^16.39^ to activate the nucleophilic water, leading to PP_i_ hydrolysis.^24–26^

However, this proposed mechanism is based on the presently available crystal structures of mPPases, which are static snap-shots and do not provide a complete picture of the intermediate steps of the catalytic mechanism. Furthermore, protein crystals represent lattices of ordered molecules that have reached equilibrium by attaining a global minimum of free-energy. Sometimes, proteins crystallize in a non-native conformation, forgoing biologically relevant interactions to establish crystallographic contacts that lower the free energy of the system.^27^ Finally, proteins in the solution are subject to thermal noise, which can have dramatic effects on the protein shape and conformation. Knowledge of the nature and magnitude of these conformational changes can be key to understanding biological mechanisms.

**FIG. 3.**
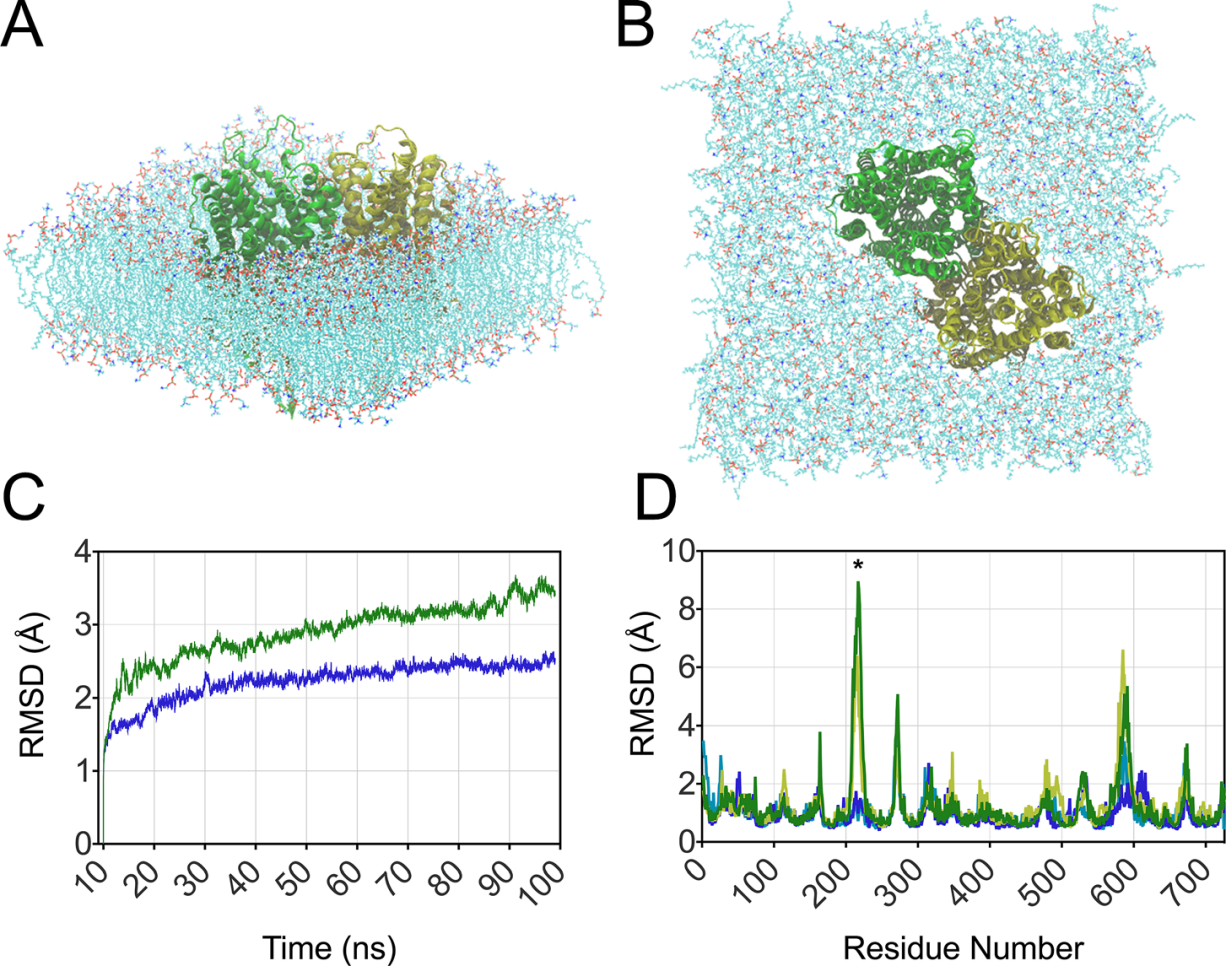
Atomistic molecular model of the TmPPase resting state in a 50% mixture of POPE:POPC lipids shown from the side (a) and top (b). The solvent molecules present in the MD simulations are not shown for clarity. (c) RMSD measured over all atoms for the resting (green) and IDP-bound (blue) states over the 100 ns trajectory. (d) Per-residue RMSD fluctuations in TmPPase for the resting protomers (green and yellow) and IDP-bound protomers (blue and cyan). The asterisk highlights loops 5–6 (residues 215–225).

To begin exploring the substrate-induced loop-closing aspects of the catalytic cycle, we performed MD simulations of TmPPase in a self-assembled lipid bi-layer. These are the first MD simulations of this class of proteins, and due to the important role of metal ions within the active site, an all-atom approach was required, which incurs substantial additional computational expense relative to united-atom (where hydrogens are ignored) or coarse-grained MD. Simulations were performed on three different models of TmPPase: the resting state TmPPase structure on to which missing loops 5–6 and 13–14 were modelled (Figs. 3(a) and 3(b)), the IDP-bound TmPPase structure, and the IDP-bound TmPPase structure from which Na^+^, IDP, and the three magnesium ions associated with IDP were removed (two magnesium ions remained at sites equivalent to the resting state TmPPase structure).

**FIG. 4.**
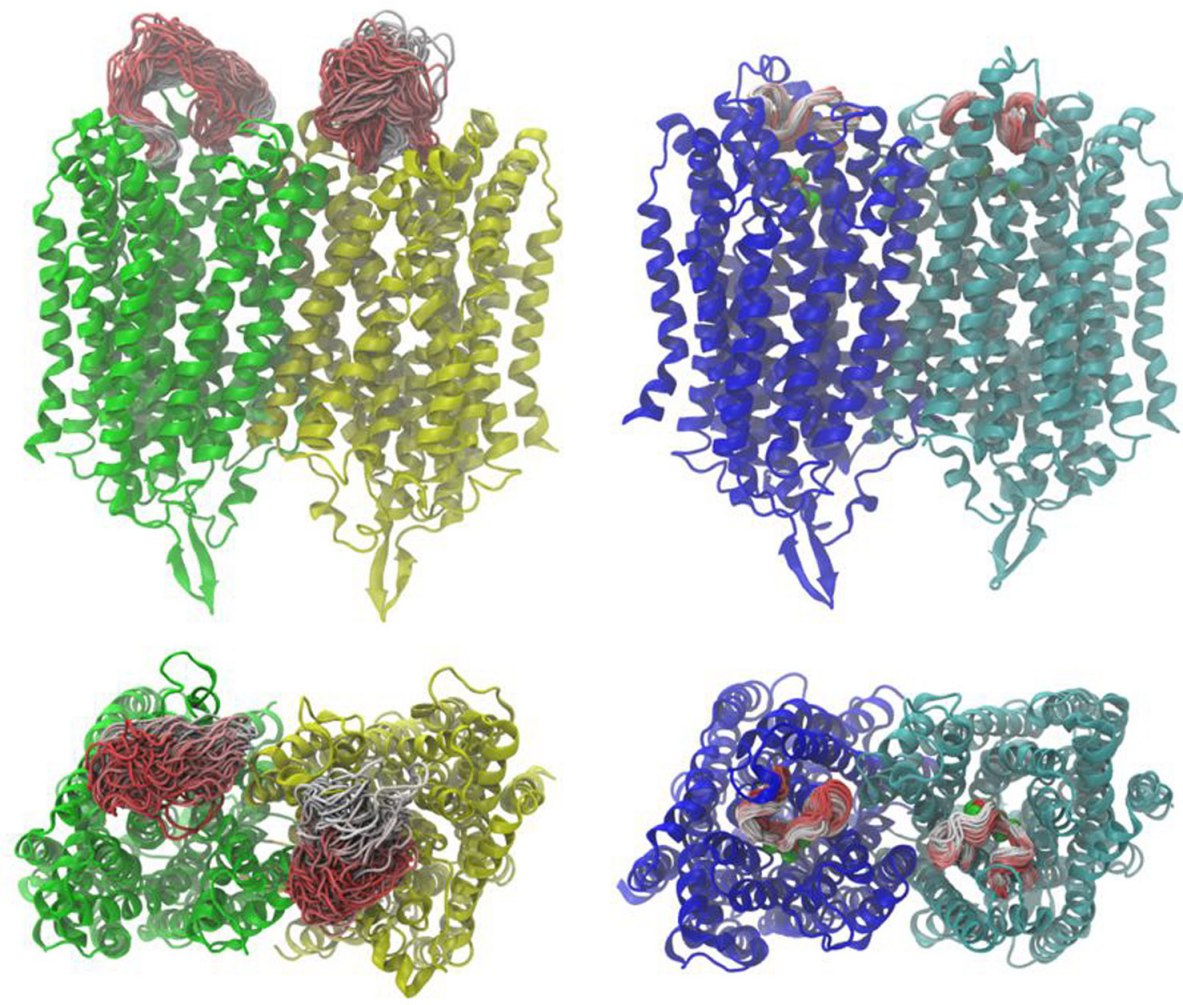
Side and top views of the resting (left, green and yellow protomers) and IDP-bound (right, blue and cyan protomers) TmPPase states, showing the range of conformations explored by loop 5–6 during the 100 ns MD. Colors in the loop indicate the position in the trajectory, ranging from red (start) to white (end). The IDP and magnesium ions are shown in space filling representation in red and green, respectively.

Over a trajectory of 100 ns, the RMSD of all protein atoms in the MD simulations of the resting and IDP-bound models was compared (Fig. 3(c)). These results showed that the resting state was significantly more dynamic than the IDP-bound state. The increase in protein flexibility in the resting state is particularly evident in loop 5–6, which is the most dynamic region, displaying dramatic fluctuations in RMSD (denoted by a star in Fig. 3(d)), with a maximum RMSD fluctuation above 8 $\overset{´}{Å}$ in one of the resting state TmPPase protomers. This is in contrast to MD simulations of the IDP-bound state that, consistent with the x-ray structural results, showed much smaller fluctuations of loop 5–6 (2 $\overset{´}{Å}$ maximum). Furthermore, the range of different conformations adopted by loop 5–6 over the course of the 100 ns MD simulation was much greater for the resting state structure compared to the IDP-bound structure (Fig. 4).

**FIG. 5.**
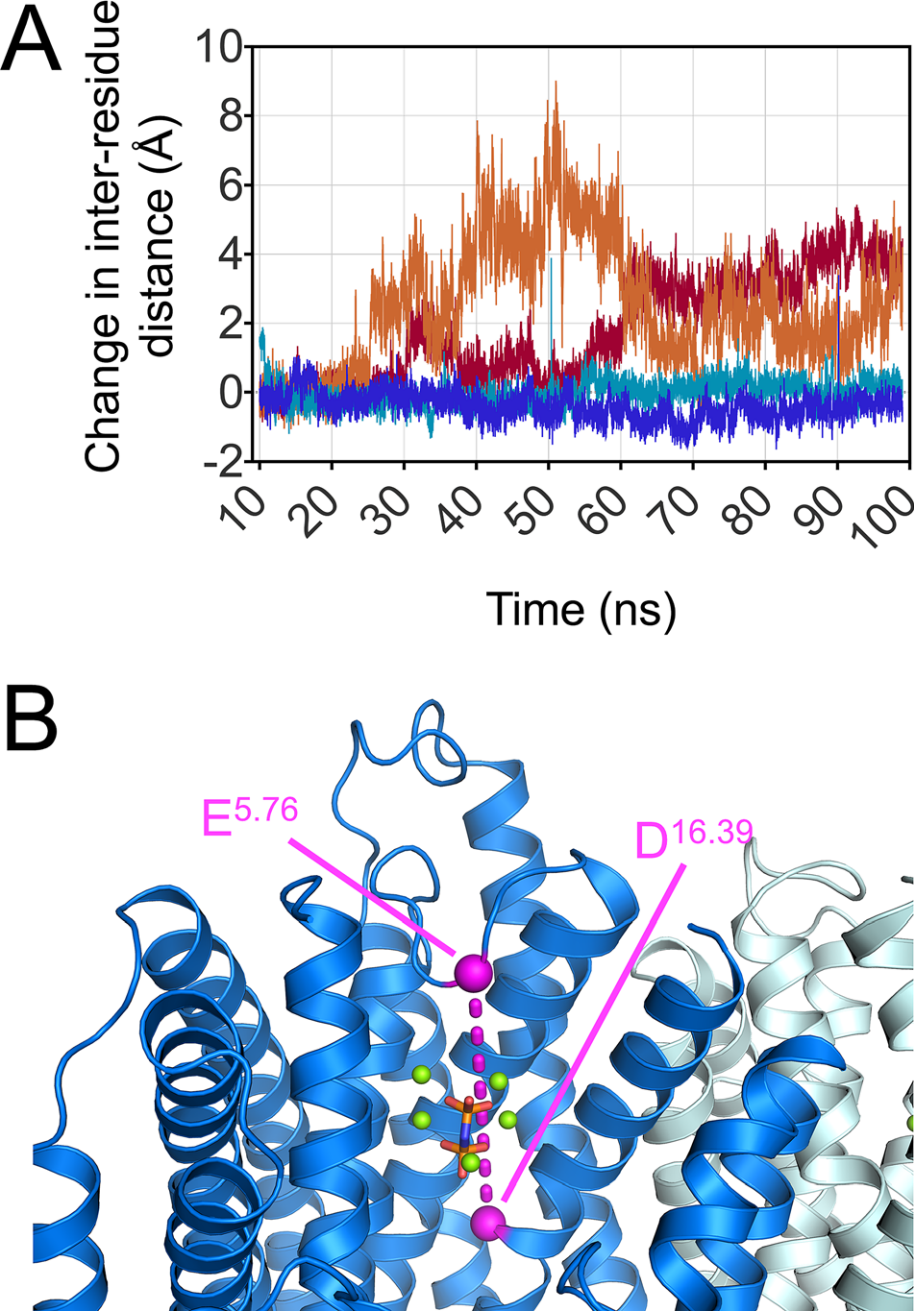
(a) Distance between Glu217 (E^5.76^) and Asp696 (D^16.39^) in each protomer when IDP is bound (blue and cyan) and when it has been removed from the pocket (red and orange). (b) IDP-bound structure of TmPPase highlighting the Glu217 (E^5.76^) and Asp696 (D^16.39^) as pink spheres. The pink dashed line represents the inter-residue distance measured in the simulations in (a). IDP is shown in red with Mg^2+^ shown as green spheres.

In addition to the difference between the resting state and the IDP-bound state, we wanted to observe what might happen to loop 5–6 once the substrates were no longer available to constrain it; this would provide information on how the substrate binds to the enzyme. We monitored the difference in distance between Glu217 (E^5.76^), which is central to loop 5–6, and Asp696 (D^16.39^), a relatively motionless residue, over the course of MD simulations of both the IDP-bound TmPPase structure and the IDP-bound TmPPase structure from which Na^+^, IDP, and the three magnesium ions associated with IDP were removed (Fig. 5). These results show that in the presence of the substrate, there is a 2 $\overset{´}{Å}$ change of distance between these two residues in either direction. However, once the substrate is removed, the loop immediately becomes more dynamic, resulting in an increase in distance between these two residues of up to 9 $\overset{´}{Å}$ over the time course (Fig. 5(a)), corresponding to an outward movement of loop 5–6 as if uncovering the active site pocket.

**FIG. 6.**
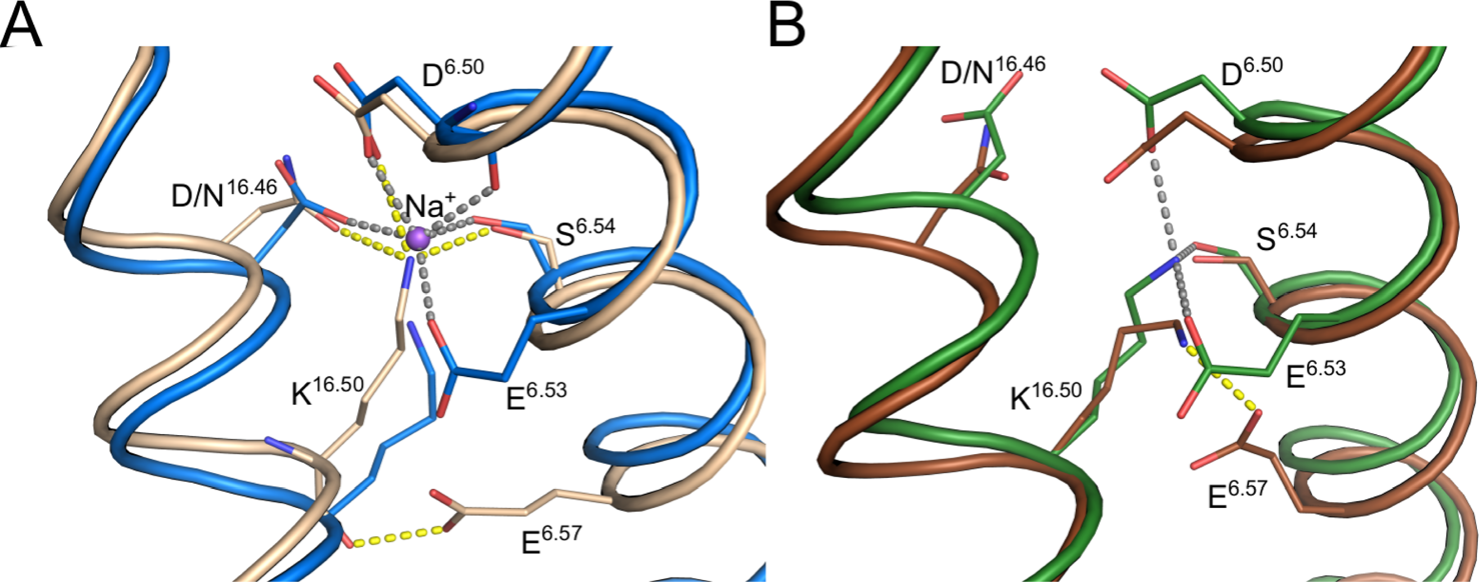
Structural overview of the ion-gate region of mPPases. (a) Comparison of the substrate-analogue-bound states of TmPPase (blue) and VrPPase (light brown) showing the coordination of the bound sodium ion (purple sphere) with neighboring residues of TmPPase and coordination of residues in the VrPPase ion gate. (b) Comparison of the resting state of TmPPase (green) with the phosphate-bound state of VrPPase (brown), highlighting the coordination of residues around the ion gate. Interactions between residues in TmPPase structures are shown in grey and VrPPase in yellow.

Taken together, these results suggest that interactions of the enzyme with IDP and Na^+^, such as IDP-Mg^2+^-H_2_O-E^5.76^, IDP-Mg^2+^-H_2_O-D^5.77^ (Fig. 2(c)), and Na^+^ with the residues E^6.53^, D^6.50^, S^6.54^, and D^16.46^ (Fig. 6(a)), as seen in the crystal structure,^31^ maintain the loop-closed, substrate-bound conformation of TmPPase. Therefore, removing the substrate results in a movement back to an open-loop, resting state-like conformation.

### B. Overview of ion pumping in mPPases

Unlike PP_i_ hydrolysis, the mechanism of ion pumping and ion selectivity is more elusive although the recently solved structures do give us some insights into possible mechanisms. The key region of the enzyme responsible for ion selectivity and pumping is the ion-gate (Fig. 6), which sits 10 $\overset{´}{Å}$ below the active site in the channel formed by the inner ring of helices. For TmPPase, we could compare all three states (TmPPase:IDP:Na, TmPPase:WO_4_, and TmPPase:Ca:Mg), but for VrPPase, only the substrate-bound and single product-bound states were available (VrPPase:IDP and VrPPase:P_i_). However, the structural architecture of the ion-gate in TmPPase:WO_4_ and the resting state TmPPase:Ca:Mg is not significantly different, and therefore, we treat TmPPase and VrPPase:P_i_ structures as equivalent in terms of interpreting differences in ion pumping (Fig. 6).

One key difference between the Na^+^-pumping TmPPase and the H^+^-pumping VrPPase in the ion-gate region is the location of the semi-conserved Glu (E^6.53^ and E^6.57^, respectively), which is one helix turn lower on the VrPPase compared to the TmPPase (Fig. 6). The importance of the semi-conserved Glu position becomes evident in the formation of the ion-gate in the resting or P_i_-bound structures. In TmPPase, the ion-gate is formed by coordination of K^16.50^ with D^6.50^ and E^6.53^, whereas a salt-bridge between K^16.50^ and E^6.57^ forms the ion-gate in VrPPase (Fig. 6(b)). The K^16.50^-E^6.57^ interaction prevents K^16.50^ from coordinating with D^6.50^. In the IDP-bound TmPPase (Fig. 6(a)), E^6.53^ coordinates the Na^+^, along with residues D^6.50^, S^6.54^, and D^16.46^. The movement of TMH 16 down 1.1 $\overset{´}{Å}$ in TmPPase moves K^16.50^ out of the Na^+^-binding pocket. This allows Na^+^ to bind, which is then coordinated by D^6.50^, E^6.53^, and S^6.54^. In this structure, the Na^+^ is still positioned above the ion-gate. In contrast, E^6.57^ (the semiconserved Glu) in the IDP-bound VrPPase structure (Fig. 6(a)) is not coordinated to K^16.50^, which is instead coordinated to D^6.50^, S^6.54^, and D/N^16.46^.^24–26^ Therefore, E^6.57^ is most likely protonated, possibly by the proton that will be pumped.

### C. Support for the transport-before-hydrolysis mechanism

The structures suggest that conformational changes upon IDP or substrate binding lead to nucleophilic water coordination and are linked to additional changes that promote ion pumping.^24–26^ Despite the logical interpretation of the crystallographic data, we do not yet know whether PP_i_ hydrolysis precedes ion pumping or whether ion pumping leads to PP_i_ hydrolysis.^6^ The former idea suggests that PP_i_ binding to mPPase results in conformational changes, such as the closing of the loops and repositioning of residues that coordinate the nucleophilic water, thus leading to activation of the nucleophilic water and PP_i_ hydrolysis. Generation of the phosphate product would then induce further conformational changes that result in ion pumping. The latter idea implies that substrate binding drives pumping, in other words, conformational changes induced by substrate binding reposition D^6.43^ so that it can coordinate the nucleophilic water alongside D^16.39^ (Fig. 2(a)), but PP_i_ is not hydrolysed at this point; indeed, it is possible that one of the aspartates is protonated. The structural changes induced by PP_i_ binding first promote ion pumping, which increases the negative charge in the active site and so causes a deprotonation of the general base D^6.43^ and/or D^16.39^. This activates the water nucleophile, leading to hydrolysis, i.e., ion pumping drives substrate hydrolysis.

Although all-atom classical MD simulations can provide insights into conformational changes of proteins in the solution over 100 ns timescales, there are still several limitations to this method. In regard to determining whether ion transport occurs before or after substrate hydrolysis, one major limitation is the inability to break covalent bonds and thus actually hydrolyze the substrate during the simulation. Therefore, complementary experimental approaches are required to fully understand the catalytic cycle of mPPases.

We have recently published data that support the latter mechanism, in which substrate binding drives ion pumping and is then followed by PP_i_ hydrolysis.^26^ We used the SURFE^2^R N1, from Nanion technologies, to directly measure the movement of charge across a membrane. In the presence of PP_i_, VrPPase translocates protons across the membrane as expected.^26^ However, in the presence of IDP, a non-hydrolyzable substrate analogue that has nitrogen in the place of the PP_i_ central oxygen atom, the ion translocation current was lower.^26^ When gramicidin was added alongside IDP, the current was decreased to the level of the phosphate control.^26^ This suggested that binding IDP by VrPPase results in a single turnover ion-pumping event. Since IDP is not converted into the product, it remains bound to VrPPase, preventing further ion translocation and thus generating a reduced current compared to PP_i_. Gramicidin permeabilizes the membrane to monovalent cations and dissipates H^+^ gradients across membranes, thus explaining the background levels of current when added with IDP.^26^

**FIG. 7.**
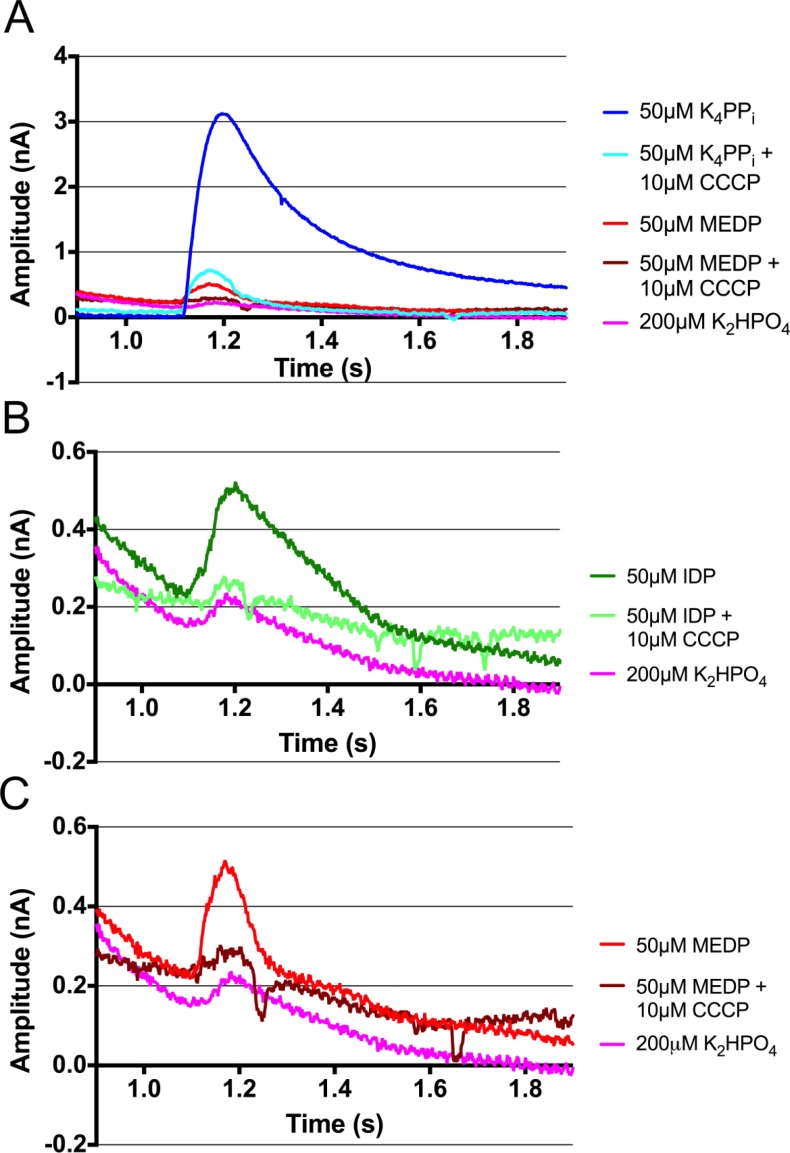
Electrometric measurements of VrPPase proteoliposomes using the Nanion SURFE^2^R N1. Currents obtained following the addition of (a) K_4_PP_i_, MEDP, K_2_HPO_4_, and all three in the presence of the protonophore: CCCP; (b) IDP, K_2_HPO_4_, and IDP with CCCP; and (c) MEDP, K_2_HPO_4_, and MEDP with CCCP. All compounds were added in the activating buffer at the 1-s time point.

We have new ion-pumping evidence that further supports the transport-before-hydrolysis model for mPPases. Carbonyl cyanide m-chlorophenyl hydrazine (CCCP) specifically permeabilizes membranes to protons. Addition of CCCP to membrane-embedded VrPPase in the presence of PP_i_ reduces the ion-pumping current from 3.10 nA to 0.65 nA above the baseline (Fig. 7(a)), illustrating that the translocation of charge across the membrane is due to H^+^ and not other monovalent ions, such as K^+^. The reduced current is not as low as the phosphate control (0.08 nA above the baseline), possibly because the rate of energy-driven proton pumping by VrPPase is greater than the CCCP-assisted diffusion rate of protons across the membrane. Replicating the previous IDP experiments shows similar results,^26^ with a small, ion-pumping peak of 0.27 nA above the baseline (Fig. 7(b)). However, replacing gramicidin with CCCP to collapse the charge separation over the membrane illustrates that IDP-bound VrPPase specifically pumps protons, as a reduced change in a current of 0.07 nA is observed. VrPPase in the presence of another substrate analogue, methylene diphosphonate (MEDP), similarly produces a change in a current of 0.29 nA (Fig. 7(c)), comparable to that observed with IDP. MEDP contains a carbon atom in place of the central oxygen atom of PP_i_ and therefore cannot be hydrolysed by mPPases. The low level of current, as seen with IDP, further supports our theory that ion pumping does not require substrate hydrolysis and instead occurs upon substrate binding. In the presence of both MEDP and CCCP, only background levels of current are measured (0.06 nA above the baseline), thus confirming the movement of charge across the membrane upon MEDP binding, and a single turn-over pumping event is due solely to H^+^.

## III. CONCLUSIONS

The numerous structures of the Na^+^-pumping TmPPase and the H^+^-pumping VrPPase determined by X-ray crystallography have provided insights into how this class of proteins hydrolyzes PP_i_ and pump ions across the membrane.^24–26^ However, to further understand the catalytic cycle, we are working towards constructing *in silico* systems that allow us to probe aspects of the mechanism that are inaccessible by X-ray crystallography, such as transition states that are energetically unstable and exist on the femtosecond timescale.^28^ Towards this goal, we have performed atomistic MD simulations of TmPPase in a self-assembled lipid bi-layer that show dramatic changes in flexibility of loop 5–6 between TmPPase in the resting state, IDP-bound state, and when IDP and Na^+^ are removed from the IDP bound state (Figs. 4–6).

Our findings suggest that tight contacts between TmPPase and the bound Mg_5_:IDP complex (Fig. 2(c)) and Na^+^ (Fig. 6(a)) help to maintain a loop-closed conformation that has previously been observed in the crystal structures, dispelling concerns that this interaction was due to a crystallographic artefact. In addition, atomistic MD simulations may be helpful in structure-based drug design, for assessing the magnitude of thermodynamically unfavourable changes both in conformational flexibility associated with binding and in the design of additional compounds with related chemical scaffolds. Since mPPases are not found in humans, targeting these proteins in human pathogens, such as protozoan parasites or *Bacteroides* species, is a viable option and would carry a reduced risk of human toxicity.^29^ One strategy would be to combine structural data with computer modelling by first identifying the binding site of the initial hit compound by X-ray crystallography. This structure can then be used as a starting point for a virtual screen to identify compounds with greater affinities. More detailed atomistic MD simulations could then be employed for the most promising leads. The top hits would then be synthesized and tested *in vitro*, before restarting the *in silico* cycle of the compound design.

Resolving the multiple steps of the mPPase reaction cycle will require employing additional approaches to overcome the limitations of classical MD simulations. Here, we have also provided further experimental evidence to support our hypothesis that substrate binding, not substrate hydrolysis, leads to ion pumping during the catalytic cycle of mPPases. MEDP, a non-hydrolysable substrate analogue, induces a small ion-pumping peak in VrPPase, similar to that observed with IDP (Figs. 7(a) and 7(c)).^26^ We interpret this as evidence that binding of a substrate analogue, which cannot be hydrolysed, leads to one cycle of H^+^-pumping in the VrPPase.^26^ Furthermore, the use of CCCP instead of gramicidin to dissipate the charge differential across the membrane proves that VrPPase is specifically pumping protons in the presence of PP_i_, IDP, and MEDP (Fig. 7), and the currents observed are not due to motion of potassium ions, as was possible in the previous gramicidin experiments.

Despite recent progress, there are still many unanswered questions. For example, what is the structural basis for ion selectivity in dual pumping mPPases, which pump both Na^+^ and H^+^? The IDP-bound structures show that the placement of the semi-conserved Glu in the VrPPase (E^6.57^) one helix turn below the position in the TmPPase (E^6.53^) destroys the Na^+^-binding pocket and that E^6.57^ in VrPPase acts as a proton acceptor at the end of the ion-channel Grotthuss chain (Fig. 6(a)).^25,26^ However, in dual-pumping mPPases, this Glu is in the same position as in Na^+^-pumping mPPases (E^6.53^). This issue is further complicated by the recent discovery that the dual-pumping mPPases evolved over two separate lineages and are therefore sub-classified into two groups. One lineage, the “true” dual-pumpers, co-transports Na^+^ and H^+^ over a range of Na^+^ concentrations, whereas the second lineage, the Na^+^-regulated dual-pumpers, loses the ability to pump H^+^ at above-physiological levels of Na^+^.^30^ Determining the molecular structure of dual-pumping mPPases in each lineage may give clues as to the flexibility of these enzymes in terms of ion selectivity, as well as the mechanism for pumping two ions. MD simulations could contribute in answering how these two lineages of dual-pumping mPPases differ in their method of ion pumping.

## IV. MATERIALS AND METHODS

### A. Ion-pumping measurements

VrPPase was reconstituted into liposomes and used to obtain electrometric measurements using the Nanion SURFE^2^R N1 following the protocol used in our previous publication.^26^ A reconstituted protein is added to proprietary sensor chips following standard protocols from Nanion technologies (details in Ref. 26). The sensor is rapidly switched between a control buffer (containing no substrate or inhibitor) and an activating buffer (containing substrate and/or inhibitor) over the course of 3 s, during which the amplitude is measured across the membrane. 50 *μ*M of the substrate (K_4_PP_i_) or inhibitors (IDP and MEDP) were used in the activating buffer in separate experimental runs, and 200 *μ*M K_2_HPO_4_ was used as a control. 10 *μ*M CCCP was used to destabilise the proton gradient. All experiments were carried out on a single sensor chip to negate the effects of inter-sensor variation, and all experiments involving CCCP were carried out after initial measurements with inhibitors to avoid residual effects of the protonophore in the membranes. The change in current was calculated by subtracting the baseline value, before the rise in the signal, from the maximal peak value.

### B. Molecular dynamics simulations

To generate the input co-ordinates for MD simulations of the resting state, loops 5–6 and 13–14 were built into the structure above the active site in an orientation that is parallel to the protomer channel, as there was no electron density reported for these loops.^24^ The missing amino acids 211–221 and 577–595 of chain A and 212–220 and 584–598 of chain B were added using software MODELLER 9v14 (Ref. 31) with default settings in the Discovery Studio 4.5 platform.^32^ Eight generated models underwent visual evaluation, and among these, the representative with loops not locating within the membrane bilayer was selected as a starting point for the simulation. This modified TmPPase resting state, the IDP-bound state, and the IDP-state after removal of the ligands (IDP, three Mg^2+^, and Na^+^) were then embedded in a membrane consisting of a 50% mixture of phosphatidylethanolamine (POPE) and phosphotidylcholine (POPC) lipids, which was of sufficient dimension to ensure at least 8 Å between the protein and the edge of the simulation box. The simulation cells were ∼135 Å^2^ in size in the plane of the membrane and contained 496 lipids in total. The protein, ligand, and lipids were modelled with the AMBER ff12SB, GAFF, and Lipid14 force fields, respectively,^33–35^ and long range electrostatics was treated with the Particle Mesh Ewald technique. The protein and membrane were then solvated with TIP3P water molecules and 0.1 M KCl using the CHARMM Molecular Modelling Builder^36^ and, after a standard equilibration procedure, were subjected to 100 ns MD at constant temperature and pressure using the Berendsen temperature and pressure coupling schemes within the AMBER suite of programs,^37^ with an MD timestep of 2 fs. Data analysis was performed using the PTRAJ module of AMBER,^38^ and trajectories were visualised using Visual Molecular Dynamics.^39^ The atomistic model built for the protein and lipid bilayer is shown for the resting state in Figs. 3(a) and 3(b).

In the first 10 ns of the MD simulations, we observed rapid and noisy fluctuations in the data as the system came to equilibrium, and for this reason, we excluded the initial 10 ns of the simulation from analysis. Consequently, in order to calculate the changes in RMSD and distances between residues, instead of taking the values of the initial frames of the MD simulation as a reference structure, an average of all states between 10 and 20 ns of the respective MD simulations was used in calculations.

## References

[1] B. S. Cooperman , A. A. Baykov , and R. Lahti , Trends Biochem. Sci. , 262 (1992). 10.1016/0968-0004(92)90406-Y 1323891

[2] R. Lahti , Microbiol. Rev. , 169 (1983). 6135978 10.1128/mr.47.2.169-178.1983 PMC281570

[3] J. H. Klemme , Z. Naturforsch. C , 544 (1976). 185830 10.1515/znc-1976-9-1011

[4] J. K. Heinonen , *Biological Role of Inorganic Pyrophosphate* ( Springer Science & Business Media, 2001).

[5] R. A. Terkeltaub , Am. J. Physiol. Cell Physiol. , C1 (2001). 10.1152/ajpcell.2001.281.1.C1 11401820

[6] T. Kajander , J. Kellosalo , and A. Goldman , FEBS Lett. , 1863 (2013). 10.1016/j.febslet.2013.05.003 23684653

[7] A. Serrano , J. R. Pérez-Castiñeira , M. Baltscheffsky *et al.*, IUBMB Life , 76 (2007). 10.1080/15216540701258132 17454298

[8] Y. M. Drozdowicz and P. A. Rea , Trends Plant Sci. , 206 (2001). 10.1016/S1360-1385(01)01923-9 11335173

[9] L. O. Björn , *Photobiology* ( Springer, 2015), pp. 207–230.

[10] F. Brini , M. Hanin , I. Mezghani *et al.*, J. Exp. Bot. , 301 (2007). 10.1093/jxb/erl251 17229760

[11] S. L. Lv , L. J. Lian , P. L. Tao *et al.*, Planta , 899 (2009). 10.1007/s00425-008-0880-4 19130078

[12] X. Li , C. Guo , J. Gu *et al.*, J. Exp. Bot. , 683 (2014). 10.1093/jxb/ert442 24474810 PMC3904725

[13] S. Park , J. Li , J. K. Pittman *et al.*, Proc. Natl. Acad. Sci. U.S.A. , 18830 (2005). 10.1073/pnas.0509512102 16361442 PMC1323196

[14] D. A. Scott , W. de Souza , M. Benchimol *et al.*, J. Biol. Chem. , 22151 (1998). 10.1074/jbc.273.34.22151 9705361

[15] N. Marchesini , S. Luo , C. Rodrigues *et al.*, Biochem. J. , 243 (2000). 10.1042/bj3470243 10727425 PMC1220954

[16] G. Lemercier , S. Dutoya , S. Luo *et al.*, J. Biol. Chem. , 37369 (2002). 10.1074/jbc.M204744200 12121996

[17] H. H. Luoto , A. A. Baykov , R. Lahti *et al.*, Proc. Natl. Acad. Sci. U.S.A. , 1255 (2013). 10.1073/pnas.1217816110 23297210 PMC3557053

[18] H. H. Luoto , E. Nordbo , A. M. Malinen *et al.*, Biochem. J. , 281 (2015). 10.1042/BJ20141434 25662511

[19] H. M. Wexler , Clin. Microbiol. Rev. , 593 (2007). 10.1128/CMR.00008-07 17934076 PMC2176045

[20] H.-S. Yoon , S.-Y. Kim , and I.-S. Kim , J. Appl. Genet. , 129 (2013). 10.1007/s13353-012-0117-x 23055406

[21] H. H. Luoto , G. A. Belogurov , A. A. Baykov *et al.*, J. Biol. Chem. , 21633 (2011). 10.1074/jbc.M111.244483 21527638 PMC3283130

[22] J. Y. Tsai , J. Kellosalo , Y. J. Sun *et al.*, Curr. Opin. Struct. Biol. , 38 (2014). 10.1016/j.sbi.2014.03.007 24768824

[23] G. A. Belogurov and R. Lahti , J. Biol. Chem. , 49651 (2002). 10.1074/jbc.M210341200 12401795

[24] J. Kellosalo , T. Kajander , K. Kogan *et al.*, Science , 473 (2012). 10.1126/science.1222505 22837527

[25] S. M. Lin , J. Y. Tsai , C. D. Hsiao *et al.*, Nature , 399 (2012). 10.1038/nature10963

[26] K.-M. Li , C. Wilkinson , J. Kellosalo *et al.*, Nat. Commun. , 13596 (2016). 10.1038/ncomms13596 27922000 PMC5150537

[27] E. Krissinel , J. Comput. Chem. , 133 (2010). 10.1002/jcc.21303 19421996

[28] S. D. Schwartz and V. L. Schramm , Nat. Chem. Biol. , 551 (2009). 10.1038/nchembio.202 19620996 PMC2859820

[29] N. R. Shah , K. Vidilaseris , H. Xhaard *et al.*, AIMS Biophys. , 171 (2016). 10.3934/biophy.2016.1.171

[30] E. Nordbo , H. H. Luoto , A. A. Baykov *et al.*, Biochem. J. , 3099 (2016). 10.1042/BCJ20160529 27487839

[31] A. Sali and T. L. Blundell , J. Mol. Biol. , 779 (1993). 10.1006/jmbi.1993.1626 8254673

[32] BIOVIA Discovery Studio, *Discovery Studio Modeling Environment* ( Dassault Systèmes, San Diego, 2016).

[33] V. Hornak , R. Abel , A. Okur *et al.*, Proteins , 712 (2006). 10.1002/prot.21123 16981200 PMC4805110

[34] J. Wang , R. M. Wolf , J. W. Caldwell , *et al.*, J. Comput. Chem. , 1157 (2004). 10.1002/jcc.20035 15116359

[35] C. J. Dickson , B. D. Madej , A. A. Skjevik *et al.*, J. Chem. Theory Comput. , 865 (2014). 10.1021/ct4010307 24803855 PMC3985482

[36] B. R. Brooks , C. L. Brooks , A. D. MacKerell *et al.*, J. Comput. Chem. , 1545 (2009). 10.1002/jcc.21287 19444816 PMC2810661

[37] D. A. Case , R. M. Betz. , W. Botello-Smith , D. S. Cerutti , T. E. Cheatham III , T. A. Darden , R. E. Duke , T. J. Giese , H. Gohlke , A. W. Goetz , N. Homeyer , S. Izadi , P. Janowski , J. Kaus , A. Kovalenko , T. S. Lee , S. LeGrand , P. Li , C. Lin , T. Luchko , R. Luo , B. Madej , D. Mermelstein , K. M. Merz , G. Monard , H. Nguyen , H. T. Nguyen , I. Omelyan , A. Onufriev , D. R. Roe , A. Roitberg , C. Sagui , C. L. Simmerling , J. Swails , R. C. Walker , J. Wang , R. M. Wolf , X. Wu , L. Xiao , D. M. York , and P. A. Kollman , *AMBER 2016* ( University of California, San Francisco, 2016).

[38] D. R. Roe and T. E. Cheatham III , J. Chem. Theory Comput. , 3084 (2013). 10.1021/ct400341p 26583988

[39] W. Humphrey , A. Dalke , and K. Schulten , J. Mol. Graphics , 33 (1996). 10.1016/0263-7855(96)00018-5 8744570

